# The impact of COVID-19 on eating disorder referrals and admissions in Waikato, New Zealand

**DOI:** 10.1186/s40337-021-00462-0

**Published:** 2021-08-28

**Authors:** Sara J. Hansen, Alice Stephan, David B. Menkes

**Affiliations:** 1 University of Auckland, Waikato Clinical Campus, Private Bag 3200, Hamilton, New Zealand; 2 Waikato District Health Board, Hamilton, New Zealand

**Keywords:** Mental health services, SARS-CoV-2, Feeding and eating disorders, Anorexia nervosa, Bulimia nervosa, New Zealand

## Abstract

**Background:**

Several countries have reported increased demand for eating disorder services during the COVID-19 pandemic, particularly for adolescents. Within New Zealand, anecdotal and media reports suggest similar changes but are limited in scope and detail. We assessed eating disorder service demand in the Waikato district in relation to the COVID-19 pandemic.

**Methods:**

We retrospectively analysed records of eating disorder admissions and referrals for both children (< 18 years) and adults (≥ 18 years) during 2019 and 2020 in the Waikato, a mixed urban–rural province in northern New Zealand (population 435,000). We analysed medical admission and outpatient referral rates, and referral acuity, in relation to the COVID-19 pandemic using Welch’s t- and chi-square tests.

**Results:**

106 medical admissions met inclusion criteria (n = 37 in 2019; 69 in 2020). Admissions for eating disorders increased markedly following nationwide lockdown in March 2020 (RR = 1.7, p = 0.01), largely driven by increases in adult admissions (RR 2.0, p = 0.005). The proportion of ‘new patient’ admissions showed comparable increases for both children (RR = 2.0, p = 0.02) and adults (RR = 2.3, p = 0.03). Following lockdown, outpatient referrals increased in acuity (RR = 1.8, p = 0.047) and volume (RR = 1.6, p = 0.076) for children but not for adults.

**Conclusions:**

Our study confirms a pandemic-related increase in demand for eating disorder services in the Waikato region of New Zealand, consistent with findings reported overseas. We observed contrasting increases in admissions for adults and outpatient referrals for children, exacerbating resource constraints for already stretched services and compromising provision of timely care.

**Plain English summary:**

The COVID-19 pandemic has been linked to increased numbers and worsening severity of eating disorders in several settings. In New Zealand, similar trends have been noted anecdotally. We assessed clinical records to calculate rates of eating disorder-related hospital admissions and outpatient referrals during 2019 and 2020. We found significant increases in hospital admissions related to COVID-19, particularly for adults, and greater proportions of both children and adults having a first-ever eating disorder-related admission. In outpatient services, young people were referred more frequently during the pandemic and were more physically unwell when referred. These results indicate increased demand for eating disorder services as a result of the pandemic and complement findings reported overseas.

**Supplementary Information:**

The online version contains supplementary material available at 10.1186/s40337-021-00462-0.

## Background

Eating disorders have reportedly increased in prevalence and severity related to the COVID-19 pandemic, in what the mainstream media has called a “crisis” or “tsunami” of demand on clinical services [1–4]. Several studies have reported stable or increasing rates of eating disorder service need while non-COVID-19 healthcare utilisation dropped during the early pandemic [5, 6]. Increased disordered eating behaviours have been documented in patients with existing eating disorders (anorexia nervosa and bulimia nervosa), and to a lesser extent in populations without any history of an eating disorder [7, 8].

Increased psychological distress has been an anticipated side-effect of the pandemic since its outset [9]. Major global changes to economic and social life, widespread uncertainty, and mortality rates in the millions have defined the COVID-19 pandemic. Worsening eating disordered behaviours have been linked to altered routine, uncertainty and lack of control, social media pressures, and loss of access to treatment support during the pandemic [10–13]. Lockdown or quarantine measures increased the magnitude of these social changes, though disruptions and distress were not confined to these periods**.**

In New Zealand, COVID-19 transmission and mortality rates have been comparatively low, attributable in part to the nation’s restrictive lockdown measures beginning in March 2020 [14]. Studies performed during the lockdown suggested increased psychological distress, particularly in those with existing mental illness [15, 16]. However, eating disorders in New Zealand have not been included in these studies. Anecdotal reports suggest a rise in eating disorder service needs related to lockdown but lack the scope to describe temporal and demographic changes.

New Zealand’s reported increase in need has occurred on a background of longstanding calls for resourcing to address unmet need for people with serious eating disorders [4, 17]. Relatively small changes in demand for eating disorder services can have significant impacts on both patients and care providers. Eating disorders have one of the highest mortality rates of mental disorders, and treatment spans many months [18]. Strained public services can result in long, and possibly unsafe, waiting lists, and diversion to the private sector for lower acuity patients who can afford it. Ensuring public services are adequately resourced to withstand fluctuations must be a priority to reduce morbidity, mortality, and inequitable access to timely care.

This study seeks to document New Zealand’s pandemic-related changes in demand for eating disorder services using rate, user characteristics, and acuity measures. Analysing these impacts may help local decision-makers prioritise interventions and resource allocation and may also prove useful for clinicians in other settings.

## Objectives

Our primary objective was to evaluate possible pandemic-related changes in the rates of medical admission and outpatient referral for eating disorders in the Waikato District Health Board (DHB) catchment. Secondary objectives were to evaluate changes to the proportion of patients admitted for the first time and the acuity of referrals to eating disorder outpatient services.

## Methods

We retrospectively reviewed all 2019 and 2020 clinical records of inpatient and outpatient eating disorder services in the Waikato DHB, a government-funded public health service covering a mixed urban–rural province in northern New Zealand (population 435,000) [19].

### Study setting

Outpatient treatment for eating disorders at Waikato DHB is provided by a specialist adult service and by specialist clinicians working within the general child and adolescent service. The adult service also provides consultation and training for four surrounding DHBs, although this role has not been considered in this study. Inpatient care for the seriously unwell occurs in general medical or paediatric wards and typically includes input from the relevant outpatient service and consultation-liaison psychiatry. Private service provision in the Waikato is very limited; a small number of clinicians provide psychological treatment for adults and older adolescents with eating disorders as part of their larger outpatient practice.

New Zealand’s lockdown alert system defined four escalating levels of social and economic restriction depending on assessed risk of COVID-19 outbreak. Levels 3 and 4 lockdowns had stringency levels of 83.3 and 96.3/100, respectively, and featured significant limitation to personal movement and interpersonal contacts and closure of non-essential businesses [20, 21]. On 23 March 2020, New Zealand moved to Level 3 for two days, before shifting to Level 4 for just over a month, returning to Level 3 on 27 April. On 13 May 2020 the alert level was reduced to Level 2, allowing the reopening of many businesses and expansion of travel and social activities. New Zealand’s largest city, Auckland, which neighbours the Waikato region, returned to a regional Level 3 lockdown from 12–30 August 2020 [22].

### Study population and data collection

Outpatient referrals were identified with a manual search of service records. Inclusion required a documented working diagnosis of an eating disorder by a specialist clinician. Inpatient admission records were identified by digital search of all hospital admissions using ICD-10 codes F50.0–50.9 and were confirmed by triangulation with outpatient and liaison psychiatry records. Inclusion required inpatient medical treatment for an eating disorder, malnourishment secondary to disordered eating, or a recognised consequence of a diagnosed eating disorder not better explained by another mechanism. Psychiatric admissions and emergency department presentations not resulting in medical admission were excluded. Diagnoses were taken from the clinical notes, which use DSM-IV classifications, and included anorexia nervosa (AN), bulimia nervosa (BN), eating disorder not otherwise specified (EDNOS) and other eating disorders (feeding and eating disorders of childhood, specific phobia related to eating, unspecified eating disorder). Binge eating presentations were not included as these patients are not funded for specialist eating disorder services in the Waikato.

Data collected from digital records included demographics (age, gender, ethnicity, diagnosis), dates of referrals and admissions, previous eating disorder inpatient treatment, and referral acuity measures. Acuity measures at the time of initial assessment included body mass index (BMI) for adults and BMI centile for children, weight loss over time, cardiovascular instability, and biochemical abnormalities [sodium, potassium, eGFR, glucose, phosphate]. Each factor was assigned a risk rating according to Junior MARSIPAN criteria [23]; BMI and weight loss over time were tailored for adults using the MARSIPAN criteria [24] and local guidelines. Detailed criteria are outlined in Additional file 1; acuity scores were determined by the single highest rated risk factor.

### Statistical analysis

Data are presented as means, proportions, or counts, with 95% confidence intervals (CIs) as appropriate. We used two-tailed Welch’s t-tests for means, Fisher’s exact test for comparison of sub-group demographic and diagnostic categories (with the Freeman-Halton extension, as required), and chi-square tests without Yates correction for dependent variable categorical outcomes. Rate ratios (RR) were calculated by dividing mean event (outpatient referral or inpatient admission) incidence per month in the pandemic exposure period by incidence in the relevant comparison period; associated p values were taken from the corresponding t-tests of mean rate differences.

We compared the years 2019 and 2020 to control for seasonal effects and also specifically assessed the association with lockdown by comparing a 15 month ‘pre-COVID-19 period’ with a 9 month ‘COVID-19 period’, beginning on the first day of restrictive lockdown in New Zealand (23 March 2020). Whilst March 2020 is included in the 15 months forming the pre-COVID-19 denominator, all events in the final week of March (23rd onwards) are counted towards the ‘COVID-19 period’ only.

## Results

### Participants

We identified 154 adult and 60 child admissions, with 77 and 29, respectively, meeting eligibility criteria as defined in Methods. For outpatients, there were 219 adult and 125 child referrals identified, with 73 and 57, respectively, meeting eligibility criteria. Additional file 2 details specific reasons for exclusion.

### Demographics

Most service users in both inpatient and outpatient settings identified as being of European descent and were female. 2019 and 2020 samples were demographically similar for both outpatients [Table 1] and inpatients [Table 2]. Overall, 5.7% of inpatients and 5.4% of outpatients identified as Māori.

**Table 1:** Inpatient demographics

	Children			Adults		
	2019	2020	*p*	2019	2020	*p*
n	13	16		24	53	
Age (mean (sd*))	15.2 (1.7)	14.8 (1.9)	0.5	29.3 (11.5)	26.3 (9.3)	0.3
Female (n (%))	13 (100%)	16 (100%)	1.0	24 (100%)	49 (92%)	0.3
Ethnicity (n (%))^†^						
European	13 (100%)	13 (81%)		23 (96%)	50 (94%)	
Māori	0	2 (13%)		1 (4%)	3 (6%)	
Other	0	1 (6%)	0.5^‡^	0	1 (2%)	1.0^‡^
Working Diagnosis (n (%))						
AN	11 (85%)	8 (50%)		21 (88%)	44 (83%)	
BN	0	0		1 (4%)	0	
EDNOS	0	2 (13%)		1 (4%)	4 (8%)	
Other	2 (15%)	6 (38%)	0.1‡	1 (4%)	5 (9%)	0.5^§^

*Standard deviation

^†^Participants identifying dual ethnicities (4 outpatients, 1 inpatient) are counted twice, as per New Zealand census guidelines [25]

^‡^Fisher’s exact test with Freeman-Halton extension (2 × 3)

^§^Fisher’s exact test with Freeman-Halton extension (2 × 4)

**Table 2:** Outpatient demographics

	Children			Adults		
	2019	2020	*p*	2019	2020	*p*
N	25	32		38	35	
Age (mean (sd*))	14.2 (2.7)	14.6 (1.9)	0.6	25.4 (11.0)	24.1 (9.7)	0.6
Female (n (%))	22 (88%)	32 (100%)	0.08	36 (95%)	34 (97%)	1.0
Ethnicity (n (%))^†^						
European	22 (88%)	28 (87%)		34 (89%)	33 (94%)	
Māori	0	1 (3%)		4 (11%)	2 (6%)	
Other	4 (16%)	3 (9%)	0.8^‡^	1 (3%)	2 (6%)	0.8^‡^
Working Diagnosis (n (%))						
AN	9 (36%)	14(43.8%)		11 (28.9%)	15 (42.9%)	
BN	3 (12%)	0		7 (18.4%)	5 (12.3%)	
EDNOS	10 (40%)	14(43.8%)		19 (50%)	13 (37.1%)	
Other	3 (12%)	4 (12.5%)	0.4^§^	1 (7.9%)	2 (5.7%)	0.7^§^

*Standard deviation

^†^Participants identifying dual ethnicities (4 outpatients, 1 inpatient) are counted twice, as per New Zealand census guidelines [25]

^‡^Fisher’s exact test with Freeman-Halton extension (2 × 3)

^§^Fisher’s exact test with Freeman-Halton extension (2 × 4)

Diagnoses remained similar between the 2019 and 2020 groups, with most inpatients having a DSM-IV diagnosis of anorexia nervosa (79.2% of the total sample) and most outpatients having a diagnosis of anorexia nervosa or EDNOS (37.7% and 42.3% of the total sample, respectively).

### Inpatient demand

**Fig. 1:**
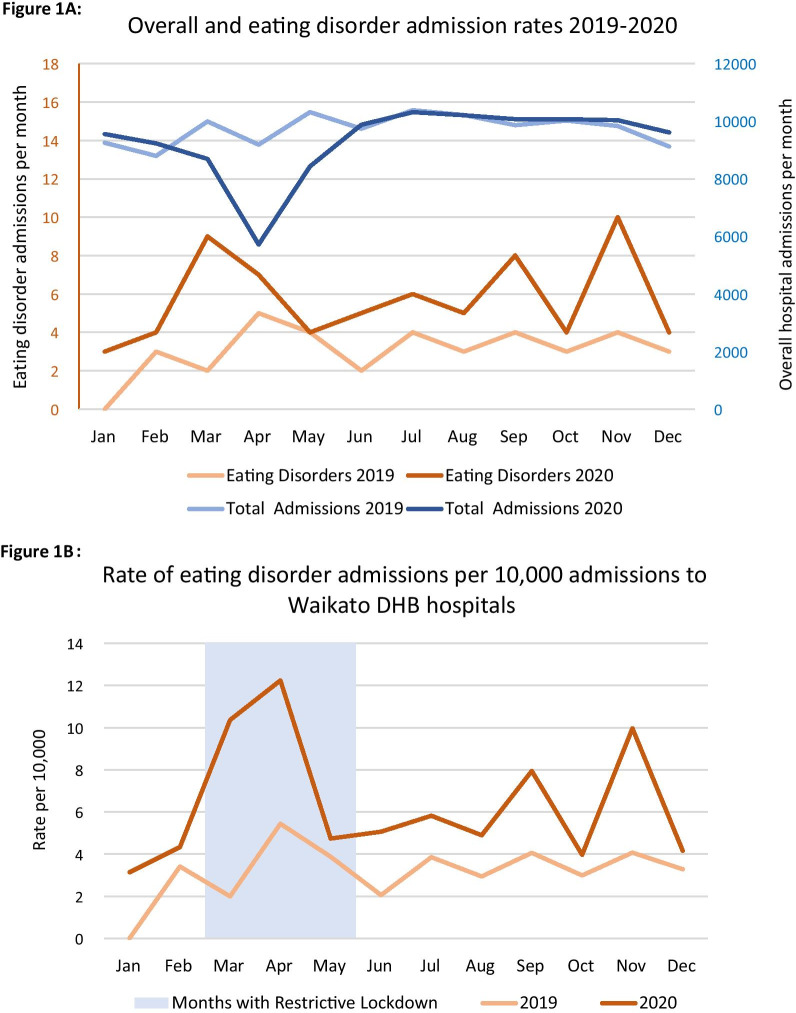
Inpatient service need. [Panel **A** Overall and eating disorder admission rates 2019–2020. Panel **B** Rate of eating disorder admissions per 10,000 admissions to Waikato DHB hospitals]. Legend: Panel **A** demonstrates a drop in the count of total admissions in April 2020, with a concurrent increase in the count of eating disorder admissions. Panel **B** shows the Rate of eating disorder admissions per 10,000 admissions to Waikato DHB hospitals; 2020 was consistently increased above 2019, with a notable spike around the commencement of lockdown (March–April)

Figure 1A demonstrates regional inpatient admission counts overall and for eating disorders. Figure 1B shows the monthly rate of eating disorder inpatient admissions per 10,000 admissions to Waikato DHB hospitals. Months affected by restrictive lockdown measures are highlighted by the shaded area, including the transition months of March and May during which onset and offset occurred partway through the month. Figure 1B demonstrates that 2020 monthly admission numbers were consistently higher than in the corresponding month in 2019, with a notable rise in March and April, around the time of initial lockdown. This is supported by Table 3, which details counts, rates, and characteristics of service use.

**Table 3:** Eating disorder service use counts, rates, and characteristics at Waikato DHB

	Pre-COVID-19	COVID-19	*p*	2019	2020	*p*
Inpatient admissions (n)						
Total	52	54		37	69	
< 18 years	17	12		13	16	
Inpatient admission rates per month (mean (95% CI))						
Total	3.5 (3.0–3.9)	6.0 (5.4–6.6)	0.01	3.1 (2.7–3.5)	5.8 (5.3–6.3)	0.002
< 18 years	1.1 (0.6–1.6)	1.3 (0.5–2.1)	0.73	1.1 (0.6 -1.6)	1.3 (0.6 -2.0)	0.62
> 18 years	2.3 (1.8–2.8)	4.7 (4.2–5.2)	0.005	2.0 (1.2–2.8)	4.4 (4.0–4.8)	0.001
‘First-ever’ inpatient admissions (n (%))						
Total	12 (23%)	24 (44%)	0.02	9 (24%)	27 (39%)	0.12
< 18 years	7 (41%)	10 (83%)	0.02	6 (46%)	11 (69%)	0.22
> 18 years	5 (14%)	14 (33%)	0.03	3 (13%)	16 (30%)	0.095
Outpatient referrals (n)						
< 18 years	29	28		25	32	
> 18 years	48	25		38	35	
Referral rates per month (mean (95% CI))						
< 18 years	1.9 (1.4–2.5)	3.1 (2.6 -3.6)	0.076	2.1 (1.5–2.7)	2.7 (2.1–3.2)	0.38
> 18 years	3.2 (2.7–3.7)	2.8 (2.0–3.6)	0.59	3.2 (2.6–3.8)	2.9 (2.3–3.5)	0.73
Referral acuity child and adolescent service (n (%))						
Low	19 (66%)	11 (39%)		18 (72%)	12 (38%)	
Medium	6 (21%)	8 (29%)		4 (16%)	10 (31%)	
High	4 (14%)	9 (32%)	0.047*	3 (12%)	10 (31%)	0.009*
Referral acuity adult service (n (%))						
Low	22 (46%)	14 (56%)		18 (47%)	18 (51%)	
Medium	11 (23%)	3 (12%)		10 (26%)	4 (11%)	
High	15 (31%)	8 (32%)	0.41*	10 (26%)	13 (37%)	0.73*

*Medium and high acuity counts were combined for a two-variable chi-square test

As shown in Table 3, the overall inpatient admission rate almost doubled in 2020 compared to 2019 (RR = 1.8, p = 0.002), and more than doubled for the adult subgroup (RR = 2.2, p = 0.001). Comparison of rates pre- and post-lockdown yields virtually identical results. The paediatric population, by contrast, did not show significant changes in admission rate. Both groups approximately doubled in the proportion of first-ever admissions during the COVID-19 period (child RR = 2.0, p = 0.02; adult RR = 2.3, p = 0.03). Within 2020, first ever admissions happened more frequently in the second half of the year (mean admissions per month January–June = 1.3; July–December = 3.2; p = 0.07). In contrast, first admissions in 2019 were balanced throughout the year (mean admissions per month January–June = 0.67; July–December = 0.83; p = 0.75). Of 24 individuals with first-ever admissions after lockdown, 4 went on to have repeat admissions during the remaining months of 2020.

### Outpatient demand

Child and adolescent outpatient services saw an average 60% increase in referrals per month following lockdown, just failing to reach statistical significance (RR 1.6, p = 0.076). Adult outpatient referral rates, by contrast, showed little change.

The child and adolescent service experienced an increased proportion of medium and high acuity referrals (RR = 1.7, p = 0.047) after lockdown, whereas adult outpatient referral acuity did not appear to change.

### Service use for patients with anorexia nervosa

[Table 4] Sub-analysis of patients with anorexia nervosa demonstrates similar results to those found overall, reflecting the substantial proportion of the sample with this diagnosis. Adult inpatients with anorexia nervosa doubled (RR = 2.1, p = 0.01) in the COVID-19 period, whereas children saw no statistically significant change. For outpatients, children had an increase in anorexia nervosa referrals (RR = 2.7, p = 0.03) while adults showed no significant change.

**Table 4:** Service use for patients with Anorexia Nervosa

	Pre-COVID-19	COVID-19	*p*	2019	2020	*p*
Inpatient admissions (n)						
Total	42	42		32	52	
< 18 years	15	9		11	7	
Inpatient admission rate per month (mean, 95% CI)						
Total	2.8 (2.4–3.2)	4.7 (4.0–5.3)	0.04	2.7 (2.2–3.2)	4.3 (3.7–4.9)	0.03
< 18 years	0.9 (0.5–1.3)	0.4 (0–1.0)	0.12	0.9 (0.4–1.4)	0.6 (0–1.3)	0.3
> 18 years	1.9 (1.4–2.4)	4 (3.4–4.6)	0.01	1.8 (1.1–2.4)	3.2 (2.7–3.6)	0.03
Outpatient referrals (n)						
Total	24	25		20	29	
< 18 years	9	14		9	14	
Outpatient referral rate per month (mean, 95% CI)						
< 18 years	0.6 (0.06–1.1)	1.6 (1.0–2.1)	0.03	0.8 (0.2–1.3)	1.2 (0.6–1.8)	0.3
> 18 years	0.9 (0.3–1.5)	1.3 (0.5–2.0)	0.7	0.9 (0.3–1.6)	1.25 (0.6–1.9)	0.5

## Discussion

The pandemic-related surge in referrals and acuity of children with eating disorders increased the strain on outpatient services and their capacity to provide timely specialist care, aligning with reports of delays locally and nationally [1, 4, 26]. This applies specifically to patients with anorexia nervosa, as well as to the overall sample of eating disordered children in the Waikato DHB.

Ours is the first study to document changes in demand for inpatient and outpatient eating disorder treatment in New Zealand. Robust, publicly available data on service use in New Zealand remain scarce. Media reports describe a doubling in paediatric admissions in Auckland, and admissions to Wellington hospital reportedly increased by 30% [1]. A private clinic in Auckland noted a 50% increase in referrals, consisting of mainly younger people, on top of a 10% year-on-year increase in service demand [3]. These reports are in keeping with the child and adolescent outpatient demand demonstrated in our study.

Our finding that paediatric admissions did not increase significantly contrasts with an Australian centre which found significant increases in admissions for anorexia nervosa in early 2020 [5]. One specialist paediatric eating disorder centre in the United Kingdom found a reduction in referrals but an increase in referral acuity, the latter consistent with our findings [27]. For adults, another British centre found stable rates of eating disorder presentations in the context of other psychiatric presentations initially dropping [6]. UK general practices saw a 40% drop in eating disorder presentations over the period from April to August [28]. Because many specialist eating disorder service referrals arise from general practice, reduced primary care service use may contribute to our finding of stable adult outpatient referrals despite increased admission rates. Additionally, for reasons of capacity over the level 3 & 4 lockdown period, only patients meeting medical admission criteria were accepted for Waikato adult outpatient services. Other referrals were put on a wait list or declined, which may mean our results underestimate adult outpatient service need during the lockdown period.

Our results are consistent with literature suggesting that patients with pre-existing eating disorders experienced clinical worsening related to COVID-19 [29–33]. In addition, our finding of increased first-ever admissions supports the idea that the general population may also be at increased risk of disordered eating behaviours as a consequence of lockdowns [7, 8]. First-ever admissions represent previously well or stable individuals becoming acutely unwell and, based on our initial experience, may result in increased service needs in the long term across both inpatient and outpatient settings. Our results provide an examination of service demand changes in a New Zealand context, and note that service needs may depend on age.

Our study does not explore potential drivers of these changes, although there have been multiple thematic correlations proposed in the literature, including social restriction, functional restriction, altered routine, altered access to treatment services, and social messages around exercise and weight gain [10–13]. While our monthly data show a spike in admissions around the time of lockdown, our lockdown-specific analysis closely resembles our calendar year-based comparison. This suggests broad impacts of the pandemic on eating disorders, beyond only lockdown stressors. For increases preceding lockdown, as noted in other centres, pandemic-related anxiety and altered routines may play a role [5]. Specific lockdown related changes, such as restricted or altered social circles, have likely contributed to the rise in need following lockdown. Deteriorations in physical or mental health may take many months to become clinically apparent, particularly for patients who were previously well or stable. This may significantly prolong the impact of the pandemic on people with eating disorders, as suggested by the increased number of first-ever inpatients admitted in the second half of 2020. Children are likely to be earlier in their disease trajectory than adults, which is reflected in the differing needs for outpatient and inpatient services by age.

We note that Māori are under-represented in both inpatient and outpatient settings, making up less than 10% of those receiving treatment for an eating disorder in the Waikato DHB, while overall 23.9% of the population is Māori, and 74.4% European [34]. The prevalence of eating disorders in Māori is thought to equal, if not surpass, the prevalence in the general population [35]. These findings are in keeping with national data indicating that Māori comprise 7% of the population receiving specialist eating disorder treatment in New Zealand [36].

There are several limitations to our study. We used a retrospective design that relied on interpreting clinical records of variable quality and detail. Our study focuses on the impact of lockdown on service use, however extensive coverage in conventional and social media ensured the pandemic was a prevalent concern in the months preceding lockdown. We hypothesise that pre-lockdown pandemic stress may have driven the slight increases in demand observed during this time, although this may be confounded by other major events in early 2020 (e.g., the catastrophic Australian wildfires) or gradual yearly increases in incidence. Ideally, comparative data would have drawn from multiple preceding years for consideration of establishing seasonal variation patterns and identifying year-on-year change. Some of the 2020 increase relative to 2019 may therefore be attributable to gradual increases in service needs, as has been noted by other providers [3]. Our inclusion criteria meant that patients who did not see a specialist for any reason were not counted. Our referral data thus probably underestimate the prevalence of need for outpatient eating disorder treatment. We used Junior MARSIPAN criteria for both adult and child risk stratification, due to the lack of accepted graded risk criteria for adults. The use of paediatric risk scales could over-estimate the risk in adult populations, but this does not alter our finding of no change in referral acuity for adults. Subgroup analyses should be interpreted with caution given small sample sizes. Differing results for lockdown and calendar year comparisons in these subgroups are more likely to reflect sampling issues than true differences.

Further research is warranted to evaluate the role of specific stressors in driving these increased needs and to define the long-term impacts of the COVID-19 pandemic on people with eating disorders. Our results also suggest ongoing barriers to care for Māori with eating disorders, warranting further exploration.

## Conclusions

Our findings indicate that patients with eating disorders in the Waikato experienced increased service needs related to the COVID-19 pandemic. Inpatient admissions for eating disorders increased markedly following lockdown, particularly for adults. The proportion of ‘new patient’ admissions for both age groups showed comparable increases. Following lockdown, outpatient referrals increased in acuity and volume for children but not for adults. This study provides a detailed single-centre perspective on a global issue, and supports findings documented in other settings. This study adds to the literature by expanding coverage of this issue to a New Zealand setting, where the pandemic featured high stringency of lockdown measures and low COVID-19 disease burden [14, 20]. While we did not explore specific drivers for increased service needs, our findings are consistent with the postulated role of lockdown-related stress and anxiety.

## Supplementary Information

**Additional file 1.** Referral Acuity Guidelines (Table). Description of thresholds used for assigning acuity ratings (Low/Medium/High) for referrals.

**Additional file 2.** Reasons for exclusion (figure). Describes specific reasons for exclusion and their proportions.

## Data Availability

The data that support the findings of this study are available from the Waikato District Health Board but restrictions apply to the availability of these data, which were used under license for the current study, and so are not publicly available. Data are however available from the authors upon reasonable request and with permission of Waikato District Health Board.

## Abbreviations

BMI: Body Mass Index
CIs: Confidence intervals
DHB: District Health Board
ICD-10: International Classification of Diseases 10
RR: Rate ratio
S.D.: Standard deviation
AN: Anorexia nervosa
BN: Bulimia nervosa
EDNOS: Eating disorder not otherwise specified
