# Supplementary material for: The impact of COVID-19 on eating disorder referrals and admissions in Waikato, New Zealand
Source: J Eat Disord. 2021 Aug 28;9:105. doi: 10.1186/s40337-021-00462-0 (PMC8397868; doi:10.1186/s40337-021-00462-0)
Supplement: Supplementary file 1 — Additional file 1. Referral Acuity Guidelines (Table). Description of thresholds used for assigning acuity ratings (Low/Medium/High) for referrals. [file 40337_2021_462_MOESM1_ESM.docx]

|  | Low Acuity | Moderate Acuity | High Acuity |
| --- | --- | --- | --- |
| Body Mass Index (BMI, >18 years) | >15 | 13-15 | <13 |
| BMI percentile (<18 years) | >2nd | 1st-2nd | <1st |
| Cardiovascular Compromise | Heart Rate >50bpm  OR Pre-syncopal symptoms with no documented abnormal orthostatic cardiovascular changes | Heart Rate 40-50bpm  OR  Single syncopal episode  OR  Orthostatic drop in blood pressure of >15mmHg systolic or >10mmHg diastolic  OR  Increase in heart rate on standing of >15bpm | Heart rate <40bpm  OR  Recurrent syncope  OR  Orthostatic hypotension of >20mmHg or more on standing  OR  Increased heart rate by >30pbm on standing |
| Weight loss over time (<18 years) | Weight loss of <10% in 3 months | Weight loss of 10-15% in 3 months | Weight loss of >15% in 3 months |
| Weight loss over time (>18 years) | 0-1.9kg weight loss within 6 weeks | 2-3.9 kg weight loss within 6 weeks | >4kg weight loss within 6 weeks |
| Biochemistry (Na, K, eGFR, Glucose, Phosphate) | No biochemical abnormalities | - | Na < 135 mmol/L  K < 3.5 mmol/L  eGFR < 75mL/min  Glucose <3.5mmol/L  Phosphate <0.7mmol/L |

**Additional File 1:** **Referral Acuity Guidelines (Table)**
