# Supplementary figures and images for: The impact of COVID-19 on eating disorder referrals and admissions in Waikato, New Zealand
Source: J Eat Disord. 2021 Aug 28;9:105. doi: 10.1186/s40337-021-00462-0 (PMC8397868; doi:10.1186/s40337-021-00462-0)

## Additional File 2: Reasons for Exclusion

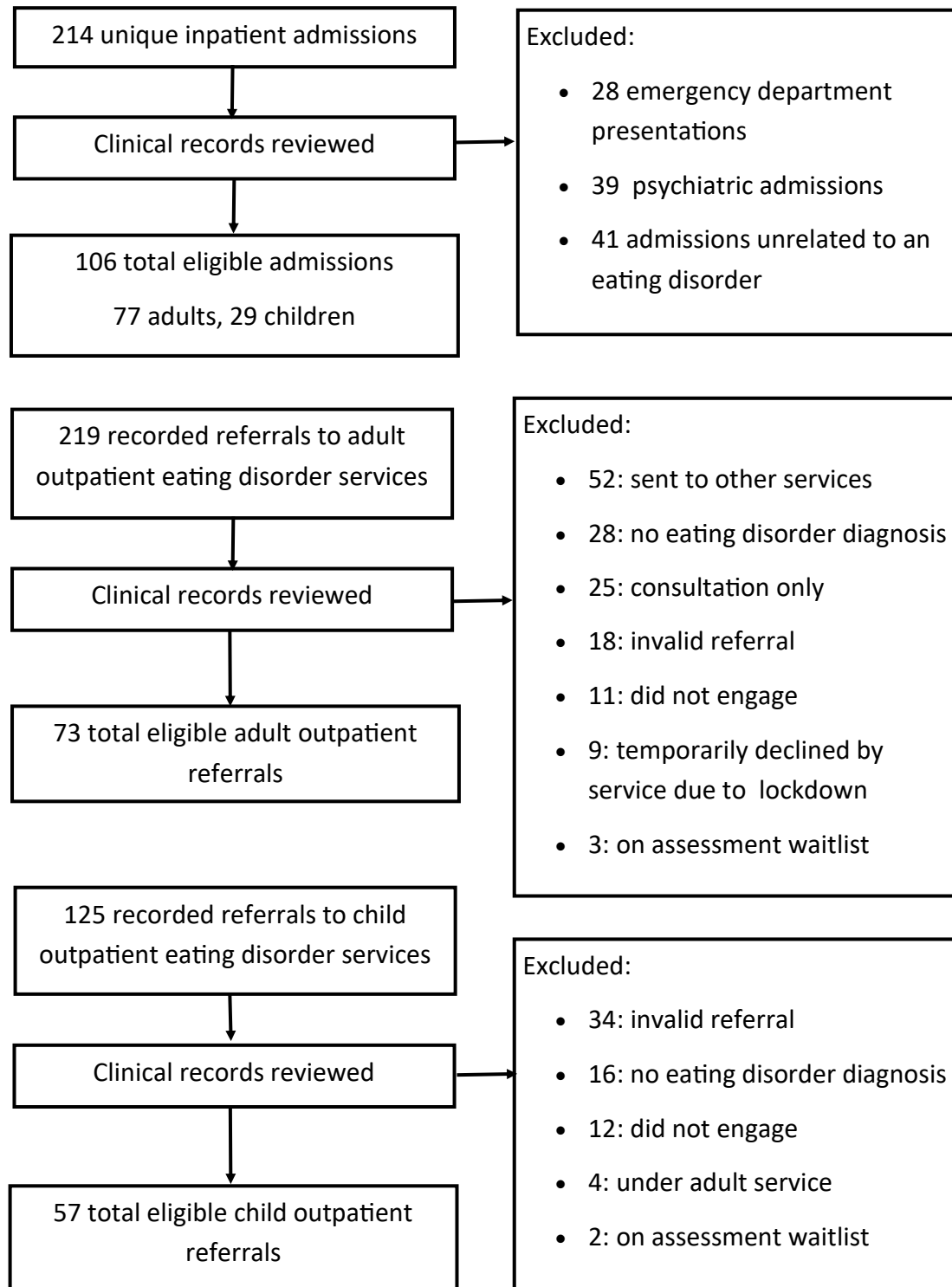

Supplement: Supplementary file 2 — Additional file 2. Reasons for exclusion (figure). Describes specific reasons for exclusion and their proportions. [file 40337_2021_462_MOESM2_ESM.pdf]
